# Spreading of Competing Information in a Network

**DOI:** 10.3390/e22101169

**Published:** 2020-10-17

**Authors:** Fabio Bagarello, Francesco Gargano, Francesco Oliveri

**Affiliations:** 1 Dipartimento di Ingegneria, Università di Palermo, Viale delle Scienze, I–90128 Palermo, Italy; fabio.bagarello@unipa.it; 2 I.N.F.N- Sezione di Napoli, 80126 Napoli, Italy; 3 Dipartimento di Scienze Matematiche e Informatiche, Scienze Fisiche e Scienze della Terra, Università di Messina, Viale F. Stagno d’Alcontres 31, I–98166 Messina, Italy; francesco.oliveri@unime.it

**Keywords:** operatorial models, (*H*,*ρ*)-induced dynamics, spreading of news, 37M05, 37N20, 47L90

## Abstract

We propose a simple approach to investigate the spreading of news in a network. In more detail, we consider two different versions of a single type of information, one of which is close to the essence of the information (and we call it *good news*), and another of which is somehow modified from some biased agent of the system (*fake news*, in our language). Good and fake news move around some agents, getting the original information and returning their own version of it to other agents of the network. Our main interest is to deduce the dynamics for such spreading, and to analyze if and under which conditions good news wins against fake news. The methodology is based on the use of ladder fermionic operators, which are quite efficient in modeling dispersion effects and interactions between the agents of the system.

## 1. Introduction

The recent rise of social media, personal blogs, and wiki-like sites has totally changed the way users relates to the diffusion of news, the users themselves being the media and distributors of news. Of course, this kind of global participatory attitude has the effect of a rapid spreading of the information, but has a main drawback—i.e., the scarce reliability of the information. The news is easily modified, distorted, or partially omitted so that much of it is just misinformation, unfounded rumors or fake news. Needless to say, the mathematical models devoted to the description of these phenomena are constantly growing in quantity. Many of them are based on concepts very often adopted in epidemiological models and graph analyses [1,2,3,4], with the goal of controlling the spreading of rumors through social media.

In this paper, we adopt an operational method based on fermionic ladder operators to describe the diffusion of news in a network of agents who are capable of receiving and transmitting information while interacting with the other agents. The underlying idea of our model is to describe a system dynamics based on the mathematical tools adopted in quantum mechanics. In particular, our framework is based on the construction of a Hamiltonian operator *H* using suitable ladder operators, and deriving the Heisenberg equations of motion to deduce the time evolution of some relevant observable parts of the system. The key observable factor in our analysis is the number operators attached to each ladder operator, from which we can obtain their mean values. This is phenomenologically interpreted as a measure of how news is considered (fake or good) by each agent. The Hamiltonian *H* contains all the operators describing the interactions occurring between the different agents of the system, in particular the diffusion of fake and good news, and the ability to modify the nature of news from good to fake and vice versa. We stress that the use of operational methods has proven successful in describing the dynamics of several macroscopic systems arising in decision making, [5], population dynamics, [6], basic cancer cell dynamics, [7,8], biological aspects of the bacterial dynamics, [9], and epigenetic evolution, [10] (see [11,12] for other fields of application).

In order to enrich the derived dynamics we shall also adopt the (H,ρ)-induced dynamics introduced in [13,14], whose approach is similar to the typical rule-oriented dynamics of the cellular automata. This approach makes it possible to introduce in the model some effects which are not easy to implement in the Hamiltonian operator.

Our main goal is to describe the typical uncertainty which accompanies the diffusion of news through non reliable agents (social media above all). We shall apply the model to two heuristic cases adopting different kind of behaviors of the agents (i.e., different *rules* ρ) which, as we shall see, can drastically change the way news is perceived.

The paper is organized as follows. In Section 2, we build the operational model for the spreading of news in a network. Since the dynamics are governed by a Hermitian time-independent quadratic Hamiltonian, the resulting dynamics are oscillatory. The dynamics are then enriched by allowing the action of some rules that modify the state of the system as a consequence of some checks on the state itself (this gives rise to a possible (H,ρ)-induced dynamic). Two main classes of rules are considered. In Section 3, two different simple applications of the general model are considered, and the results of the numerical simulations are discussed. Section 4 contains some final remarks. Additionally, for the reader’s convenience, we give a simple sketch of the (H,ρ)-induced dynamics approach in Appendix A.

## 2. The Model and Its Dynamics

As discussed in the Introduction, our main interest is to deduce a reasonable dynamical behavior for a single *bit of information*, the news N, that, depending on who is *processing* it, can be transmitted in a rather neutral way (*good news*, meaning with this that what is transmitted is just the original N), or it can be somehow distorted, because of the agent’s convenience, ignorance or for other reasons. This is what we call *fake news*. Suppose we have *N* agents, creating, receiving and transmitting N. They are labeled by indices α=1,…,N. We treat the various agents, $A_{\alpha }$, as different cells of a network S. Two cells α and β are neighboring if the agents $A_{\alpha }$ and $A_{\beta }$ have a direct link to interchange information. The cells are far away, in our picture of S, if $A_{\alpha }$ is connected to $A_{\beta }$ by means of intermediate agents. For each α we introduce two families of fermionic operators, $f_{\alpha }$ and $g_{\alpha }$, satisfying the camonical anticommutation rules (CAR)

(1) $$\left\{f_{\alpha },f_{\beta }^{†}\right\}=\left\{g_{\alpha },g_{\beta }^{†}\right\}=\delta_{\alpha ,\beta }I,\;I\;\mathrm{identity}\;\mathrm{operator},$$

with all the other anti-commutators trivial. In particular $f_{\alpha }^{2}=g_{\alpha }^{2}=0$, and $\{f_{\alpha }^{♯},g_{\beta }^{♯}\}=0$, where $x^{♯}=x$ or $x^{†}$. We also define the number operators ${\hat{F}}_{\alpha }=f_{\alpha }^{†}f_{\alpha }$ and ${\hat{G}}_{\alpha }=g_{\alpha }^{†}g_{\alpha }$. For each α we also construct a four-dimensional Hilbert space introducing first the vacua of $f_{\alpha }$ and $g_{\alpha }$. These are two vectors, $e_{\alpha ,0}^{\left(f\right)}$ and $e_{\alpha ,0}^{\left(g\right)}$ satisfying the equations $f_{\alpha }\;e_{\alpha ,0}^{\left(f\right)}=g_{\alpha }\;e_{\alpha ,0}^{\left(g\right)}=0$. Then, we define $e_{\alpha ,1}^{\left(f\right)}=f_{\alpha }^{†}e_{\alpha ,0}^{\left(f\right)}$, $e_{\alpha ,1}^{\left(g\right)}=g_{\alpha }^{†}e_{\alpha ,0}^{\left(g\right)}$, and

(2) $$\varphi_{\alpha :n_{f},n_{g}}=e_{\alpha ,n_{f}}^{\left(f\right)}⊗e_{\alpha ,n_{g}}^{\left(g\right)},$$

where $n_{f},n_{g}=0,1$. The set $F_{\varphi }\left(\alpha \right)=\left\{\varphi_{\alpha :n_{f},n_{g}}\right\}$ is an orthonormal basis in a Hilbert space $H_{\alpha }$, which is a four-dimensional vector space endowed with scalar product ${\langle .,.\rangle }_{\alpha }$:

$${\langle \varphi_{\alpha :n_{f},n_{g}},\varphi_{\alpha :m_{f},m_{g}}\rangle }_{\alpha }=\delta_{n_{f},m_{f}}\delta_{n_{g},m_{g}}.$$

Moreover,

(3) $${\hat{F}}_{\alpha }\varphi_{\alpha :n_{f},n_{g}}=n_{f}\;\varphi_{\alpha :n_{f},n_{g}},\;{\hat{G}}_{\alpha }\varphi_{\alpha :n_{f},n_{g}}=n_{g}\;\varphi_{\alpha :n_{f},n_{g}}.$$

Similarly to what is done in several contexts [11,12], we associate the following meaning to each $\varphi_{\alpha :n_{f},n_{g}}$: if the system S in α is described by the vector $\varphi_{\alpha :0,0}$, then N has not reached $A_{\alpha }$, in any of its form. If it is described by $\varphi_{\alpha :1,0}$, then the fake version of N has reached $A_{\alpha }$, while $A_{\alpha }$ was reached by its good version if the vector is $\varphi_{\alpha :0,1}$. Finally, both versions of N have reached $A_{\alpha }$ if S is described by $\varphi_{\alpha :1,1}$.

It is clear that each vector $f_{\alpha }\in H_{\alpha }$ is a linear combination of the $\varphi_{\alpha :n_{f},n_{g}}$. Now we can consider $H={⊗}_{\alpha }H_{\alpha }$, the Hilbert space of S, with scalar product

$$\langle f,g\rangle =\prod_{\alpha }{\langle f_{\alpha },g_{\alpha }\rangle }_{\alpha },$$

for each $f={⊗}_{\alpha }f_{\alpha }$ and $g={⊗}_{\alpha }g_{\alpha }$. Each operator ${\hat{X}}_{\alpha }$ acting on $H_{\alpha }$ can be extended to all of H by identifying ${\hat{X}}_{\alpha }$ with ${\hat{X}}_{\alpha }⊗\left({⊗}_{\beta \neq \alpha }I_{\beta }\right)$, where $I_{\beta }$ is the identity operator on $H_{\beta }$. The initial state of S is described the the following vector on H:

(4) $$\Psi_{n,m}={⊗}_{\alpha }\varphi_{\alpha :n_{\alpha },m_{\alpha }},$$

where $n=(n_{1},n_{2},\ldots ,n_{N})$, $m=(m_{1},m_{2},\ldots ,m_{N})$. The knowledge of $\Psi_{n,m}$ allows us to deduce if, and which kind of, information has reached any agent $A_{\alpha }$ of the system. In fact, $\Psi_{n,m}$ represents the initial diffusion of N all along the network R. In the following section, we will discuss how we can give this dynamic to S, and the effect of this dynamic.

### 2.1. The Hamiltonian and Its Effect

We first consider a time interval, [0,T], in which the dynamical behavior of S is only driven by a certain operator, the Hamiltonian *H* of the system [11,12], describing the main interactions occurring in S during that interval. In particular, we assume the following form for *H*:

(5) $$\left\{\begin{array}{cc} H & =H_{0}+H_{I},\;\;\mathrm{with}\;\\ H_{0} & =\sum_{\alpha }\omega_{f,\alpha }{\hat{F}}_{\alpha }+\sum_{\alpha }\omega_{g,\alpha }{\hat{G}}_{\alpha },\\ H_{I} & =\sum_{\alpha ,\beta }\;p_{\alpha ,\beta }^{\left(f\right)}\left(f_{\alpha }f_{\beta }^{†}+f_{\beta }f_{\alpha }^{†}\right)+\sum_{\alpha ,\beta }\;p_{\alpha ,\beta }^{\left(g\right)}\left(g_{\alpha }g_{\beta }^{†}+g_{\beta }g_{\alpha }^{†}\right)+\sum_{\alpha }\;\lambda_{\alpha }\left(f_{\alpha }g_{\alpha }^{†}+g_{\alpha }f_{\alpha }^{†}\right). \end{array}\right.$$

The first term, $H_{0}$, describes the inertia of the various agents, i.e., their tendency to keep, or change, the original message they have received [11,12]. The first two terms in $H_{I}$ describe how N is moving in the network R. In particular, the first term is a diffusion term for fake news, while the second one describes the diffusion of good news. The third term describes a possible interaction between fake and good news in each cell α: the nature of N, as perceived by the agent $A_{\alpha }$, can change during the time evolution. Of course, if $\lambda_{\alpha }=0$ for some α, the news is left unchanged by agent $A_{\alpha }$. We refer to [11,12] for several considerations on this approach and on the use of Hamiltonians in the analysis of similar systems, and in the rationale for the interpretation of the various terms in *H*.

Some remarks are in order. *H* is Hermitian; this is connected to the fact that news can move along R going back and forth from, say, $A_{\alpha }$ to $A_{\beta }$ and then back to $A_{\alpha }$ again. This is clearly possible for information which can be easily be reflected to the original source from the receiver. The coefficients $p_{\alpha ,\beta }^{\left(f,g\right)}$ in $H_{I}$ are *diffusion coefficients* for the two classes of news. We assume that they are all real and symmetric ($p_{\alpha ,\beta }^{\left(f,g\right)}=p_{\beta ,\alpha }^{\left(f,g\right)}$); moreover, we take $p_{\alpha ,\alpha }^{\left(f,g\right)}=0$.

A reasonable assumption on the diffusion coefficients is that

$$\sum_{\alpha ,\beta }p_{\alpha ,\beta }^{\left(f\right)}>\sum_{\alpha ,\beta }p_{\alpha ,\beta }^{\left(g\right)},$$

since in everyday life we usually observe that fake news diffused much faster than good news. One of the main interests in this paper is to understand how it is possible to slow down the diffusion of fake news, increasing that of good news.

We now use *H*, and the CAR in (1), to deduce the equations of motion for the ladder operators and the mean values of their related number operators. In particular, using the Heisenberg approach, we have

(6) $$\left\{\begin{array}{cc} \; & {\dot{f}}_{\alpha }\left(t\right)=-i\omega_{f,\alpha }\;f_{\alpha }\left(t\right)+2i\sum_{\beta }p_{\alpha ,\beta }^{\left(f\right)}f_{\beta }\left(t\right)+i\lambda_{\alpha }g_{\alpha }\left(t\right),\\ \; & {\dot{g}}_{\alpha }\left(t\right)=-i\omega_{g,\alpha }\;g_{\alpha }\left(t\right)+2i\sum_{\beta }p_{\alpha ,\beta }^{\left(g\right)}g_{\beta }\left(t\right)+i\lambda_{\alpha }f_{\alpha }\left(t\right), \end{array}\right.$$

where α=1,2,…,N. This is a closed system of linear, operator-valued, differential equations which can be easily solved. In fact, denoting with X(t) the 2N-column vectors whose transpose is

$$X{\left(t\right)}^{T}=\left(f_{1}\left(t\right),f_{2}\left(t\right),\ldots ,f_{N}\left(t\right),g_{1}\left(t\right),g_{2}\left(t\right),\ldots ,g_{N}\left(t\right)\right),$$

and introducing the following Hermitian 2N×2N matrix *V*,

$$V=\left(\begin{array}{cccccccccccc} -\omega_{f,1} & 2p_{1,2}^{\left(f\right)} & 2p_{1,3}^{\left(f\right)} & . & . & 2p_{1,N}^{\left(f\right)} & \lambda_{1} & 0 & 0 & . & . & 0\\ 2p_{1,2}^{\left(f\right)} & -\omega_{f,2} & 2p_{2,3}^{\left(f\right)} & . & . & 2p_{2,N}^{\left(f\right)} & 0 & \lambda_{2} & 0 & . & . & 0\\ 2p_{1,3}^{\left(f\right)} & 2p_{3,2}^{\left(f\right)} & -\omega_{f,3} & . & . & 2p_{3,N}^{\left(f\right)} & 0 & 0 & \lambda_{3} & . & . & 0\\ . & . & . & . & . & . & . & . & . & . & . & .\\ . & . & . & . & . & . & . & . & . & . & . & .\\ 2p_{1,N}^{\left(f\right)} & 2p_{2,N}^{\left(f\right)} & 2p_{3,N}^{\left(f\right)} & . & . & -\omega_{f,N} & 0 & 0 & . & . & . & \lambda_{N}\\ \lambda_{1} & 0 & 0 & . & . & 0 & -\omega_{g,1} & 2p_{1,2}^{\left(g\right)} & 2p_{1,3}^{\left(g\right)} & . & . & 2p_{1,N}^{\left(g\right)}\\ 0 & \lambda_{2} & 0 & . & . & 0 & 2p_{1,2}^{\left(g\right)} & -\omega_{g,2} & 2p_{2,3}^{\left(g\right)} & . & . & 2p_{2,N}^{\left(g\right)}\\ 0 & 0 & \lambda_{3} & . & . & 0 & 2p_{1,3}^{\left(g\right)} & 2p_{3,2}^{\left(g\right)} & -\omega_{g,3} & . & . & 2p_{3,N}^{\left(g\right)}\\ . & . & . & . & . & . & . & . & . & . & . & .\\ . & . & . & . & . & . & . & . & . & . & . & .\\ 0 & 0 & . & . & . & \lambda_{N} & 2p_{1,N}^{\left(g\right)} & 2p_{2,N}^{\left(g\right)} & 2p_{3,N}^{\left(g\right)} & . & . & -\omega_{g,N} \end{array}\right),$$

system (6) can be rewritten as

(7) $$\dot{X}\left(t\right)=iVX\left(t\right),$$

whereupon the solution is

(8) X(t)=exp(iVt)X(0),

X(0) being the initial condition. In writing the explicit form of *V* we have used the equality $p_{\alpha ,\beta }^{\left(f,g\right)}\;=\;p_{\beta ,\alpha }^{\left(f,g\right)}$. Let us call $v_{i,j}\left(t\right)={\left(\exp(iVt)\right)}_{i,j}$, i,j=1,2,…,2N, and let $E=\{e_{j},\;j=1,2,\ldots ,2N\}$ be the canonical orthonormal basis in $H_{2N}\equiv C^{2N}$, endowed with scalar product ${\langle \cdot ,\cdot \rangle }_{2N}$. Then we have

(9) $$f_{\alpha }\left(t\right)={\langle e_{\alpha },X\left(t\right)\rangle }_{2N},\;g_{\alpha }\left(t\right)={\langle e_{N+\alpha },X\left(t\right)\rangle }_{2N},$$

α=1,2,…,N. If we call $F_{\beta }^{0}$ and $G_{\beta }^{0}$ the mean value of ${\hat{F}}_{\beta }$ and ${\hat{G}}_{\beta }$ on the vector $\varphi_{\beta :n_{\beta },m_{\beta }}$ at t=0,

$$F_{\beta }^{0}={\langle \varphi_{\beta :n_{\beta },m_{\beta }},{\hat{F}}_{\beta }\varphi_{\beta :n_{\beta },m_{\beta }}\rangle }_{\beta },\;G_{\beta }^{0}={\langle \varphi_{\beta :n_{\beta },m_{\beta }},{\hat{G}}_{\beta }\varphi_{\beta :n_{\beta },m_{\beta }}\rangle }_{\beta },$$

then, calling

(10) $$\begin{array}{cc} \; & F_{\alpha }\left(t\right)=\langle \Psi_{n,m},{\hat{F}}_{\alpha }\left(t\right)\Psi_{n,m}\rangle =\langle \Psi_{n,m},f_{\alpha }^{†}\left(t\right)f_{\alpha }\left(t\right)\Psi_{n,m}\rangle \\ \; & G_{\alpha }\left(t\right)=\langle \Psi_{n,m},{\hat{G}}_{\alpha }\left(t\right)\Psi_{n,m}\rangle =\langle \Psi_{n,m},g_{\alpha }^{†}\left(t\right)g_{\alpha }\left(t\right)\Psi_{n,m}\rangle , \end{array}$$

we get

(11) $$\begin{array}{cc} \; & F_{\alpha }\left(t\right)=\sum_{\beta =1}^{N}\left(|v_{\alpha ,\beta }{\left(t\right)|}^{2}F_{\beta }^{0}+{\left|v_{\alpha ,\beta +N}\left(t\right)\right|}^{2}G_{\beta }^{0}\right),\\ \; & G_{\alpha }\left(t\right)=\sum_{\beta =1}^{N}\left(|v_{\alpha +N,\beta }{\left(t\right)|}^{2}F_{\beta }^{0}+{\left|v_{\alpha +N,\beta +N}\left(t\right)\right|}^{2}G_{\beta }^{0}\right), \end{array}$$

α=1,2,…,N. From these functions we define the following mean values:

(12) $$F\left(t\right)=\frac{1}{N}\;\sum_{\alpha =1}^{N}F_{\alpha }\left(t\right),\;G\left(t\right)=\frac{1}{N}\;\sum_{\alpha =1}^{N}G_{\alpha }\left(t\right),$$

that we interpret as the time evolution of the *global* mean values of fake and good news in R. On the other hand, $F_{\alpha }\left(t\right)$ and $G_{\alpha }\left(t\right)$ are their *local* counterparts. Notice that, because of the fermionic nature of the operators involved, we have $F_{\alpha }\left(t\right),G_{\alpha }\left(t\right)\in \left[0,1\right]$, and therefore F(t),G(t)∈[0,1] as well.

The various mean values $F_{\alpha }\left(t\right),G_{\alpha }\left(t\right)$ are the main functions we are interested to. In fact, they can be phenomenologically interpreted as the intensities of fake news and good news, respectively, the agents perceive. In the case $F_{\alpha }\left(t\right)\gg G_{\alpha }\left(t\right)$ ($F_{\alpha }\left(t\right)\ll G_{\alpha }\left(t\right)$, respectively), there is no doubt the agent $A_{\alpha }$ perceives news as fake (good, respectively), whereas the condition $F_{\alpha }\left(t\right)\approx G_{\alpha }\left(t\right)$ reflects the uncertainty about the reliability of news. Consequently, F(t),G(t) are a sort of global intensity of how news is perceived by the whole system.

**Remark** **1.** *If we introduce the operator ${\hat{N}}_{tot}=\sum_{\alpha =1}^{N}\left({\hat{F}}_{\alpha }+{\hat{G}}_{\alpha }\right)$, it is possible to check that $[H,{\hat{N}}_{tot}]=0$. This implies that, when the dynamics is only driven by H (as in this section), ${\hat{N}}_{tot}$ stays constant in time, and, consequently, also F(t)+G(t). This means that when news N moves around R, its nature can be modified during the time evolution, but the overall amount of fake and good news stays unchanged. This is reasonable since, if news is globally considered a fake, F(t) increases while G(t) decreases, and vice versa if news is considered good. In any case, in the next section we will propose a strategy, already used in other contexts, breaking this feature of the model.*

### 2.2. The Effect of the Rule

In [13], we have proposed a general strategy to modify the dynamics of a system S when some of the phenomena occurring during its time evolution cannot be encoded completely in a (Hermitian) Hamiltonian. This is particularly interesting when, from time to time, some checks are performed on S and on some of the quantities describing S, whereupon the Hamiltonian, or the state of the system, are modified accordingly to the result of the checks. We refer to [12,13,14] for a detailed discussion of this procedure, and for several applications—in any case, a short review of this idea is given in Appendix A. We have called the dynamics deduced in this way (H,ρ)-dynamics, since they are based on the idea that, while *H* drives the dynamics in the time intervals [0,T[, [T,2T[, [2T,3T[, and so on, at times *T*, 2T, 3T some transition may occur in the system due to the fact that we perform a periodic check on S, and we modify some ingredients of S accordingly. This is what we call *the rule* ρ. Then, the evolution starts again, but with these modified values of parameters, states, and so on, depending on the nature of ρ.

The quantity *T* should be therefore considered as the time taken by the agents to interact with the other agents of S before the status of N could be changed. In our system the explicit form of the rule is based on the following idea: let us consider a given cell α, and let $\Gamma_{\alpha }$ be the set of all the cells which are connected to the cell α, with the possible inclusion of the cell α itself. Let $w_{\alpha }^{f,g}\left(\gamma \right)$ be the weights assigned to the cells γ in $\Gamma_{\alpha }$ according to the nature of news, as we clarify below. We set

(13) $$F_{\Gamma_{\alpha }}\left(T\right):=\frac{1}{W_{\alpha }^{f}}\sum_{\gamma \in \Gamma_{\alpha }}w_{\alpha }^{f}\left(\gamma \right)F_{\gamma }\left(T\right),\;G_{\Gamma_{\alpha }}\left(T\right):=\frac{1}{W_{\alpha }^{g}}\sum_{\gamma \in \Gamma_{\alpha }}w_{\alpha }^{g}\left(\gamma \right)G_{\gamma }\left(T\right),$$

where $W_{\alpha }^{f,g}=\sum_{\gamma \in \Gamma_{\alpha }}w_{\alpha }^{f,g}\left(\gamma \right)$ are the sums of the weights, and let

(14) $$\Delta_{\Gamma_{\alpha }}\left(T\right)=F_{\Gamma_{\alpha }}\left(T\right)-G_{\Gamma_{\alpha }}\left(T\right)$$

be their difference. If $\Delta_{\Gamma_{\alpha }}\left(T\right)>0$ then, in our model, this is seen as evidence of the fact that, at t=T, $A_{\alpha }$ is surrounded by agents distributing more fake news rather than good news. The opposite happens if $\Delta_{\Gamma_{\alpha }}\left(T\right)<0$, while the two versions of N are balanced around $A_{\alpha }$ if $\Delta_{\Gamma_{\alpha }}\left(T\right)=0$. Of course, this interpretation is also related to the explicit choice of the weights.

These basic ingredients can be used now to set up several interesting forms of rules. In the following section, we will consider two different possibilities, described below.

#### 2.2.1. Rule $\rho_{1}$: Strongly Passive

In this first case, we assume that $\Gamma_{\alpha }$ does not contain the cell α. Rule $\rho_{1}$ acts on the state of the system in the following (very easy) way: suppose $\Delta_{\Gamma_{\alpha }}\left(T\right)>0$. Then, the status of news of the agent $A_{\alpha }$, independently of its own status (i.e., independently of the values of $F_{\alpha }\left(T_{-}\right)$ and $G_{\alpha }\left(T_{-}\right)$), is changed to $\varphi_{\alpha :1,0}$: maximum fake and minimum good. On the contrary, if $\Delta_{\Gamma_{\alpha }}\left(T\right)<0$, the state is changed to $\varphi_{\alpha :0,1}$, independently of the values of $F_{\alpha }\left(T_{-}\right)$ and $G_{\alpha }\left(T_{-}\right)$. We use $T_{-}$ to stress that we are approaching *T* from below, and that in *T* the rule is applied. Hence, the values of, say $F_{\alpha }\left(T_{-}\right)$ and $F_{\alpha }\left(T\right)$ are, in general, different. Of course, it may also happen that $\Delta_{\Gamma_{\alpha }}\left(T\right)=0$. When this happens, we let the system evolve up to 2T without changing anything, and then we apply the rule. Of course, this check must be performed for each α. Then, what we are essentially doing, is replacing the vector $\Psi_{n,m}$ in (4) with a new one, $\Psi_{n^{\left(1\right)},m^{\left(1\right)}}$, of the same kind but with (n,m) replaced by the new vectors $\left(n^{\left(1\right)},m^{\left(1\right)}\right)$, deduced by the action of the rules. In the interval [T,2T[ the evolution is again driven by the same *H*, and at t=2T the rule is applied once again, changing again the state from $\Psi_{n^{\left(1\right)},m^{\left(1\right)}}$ to $\Psi_{n^{\left(2\right)},m^{\left(2\right)}}$ and so on. The reason why this rule is referred to as strongly passive should be clear: the original (i.e., for t=T) status of $A_{\alpha }$ plays no role in what happens to S after the check. $A_{\alpha }$ only looks at what is happening in $\Gamma_{\alpha }$.

#### 2.2.2. Rule $\rho_{2}$: Active

The rule $\rho_{2}$ is a slight variation of the previous one. In this case we suppose that $\Gamma_{\alpha }$ contains the α, and hence the status of the agent $A_{\alpha }$ depends on its status too. This is a small mathematical difference, with respect to $\rho_{1}$, but it has a deep meaning. As before, the agent $A_{\alpha }$ changes the status to $\varphi_{\alpha :1,0}$ or $\varphi_{\alpha :0,1}$ according the value of $\Delta_{\Gamma_{\alpha }}\left(T\right)$.

## 3. Applications

In this section, we present some numerical applications of the model we have constructed. The main focus is to describe different kinds of diffusion dynamics and interactions between agents together with different application of the rules. We consider two simple situations; more realistic simulations are postponed to a forthcoming paper.

### 3.1. A Simple Network with Three Agents

**Figure 1:**
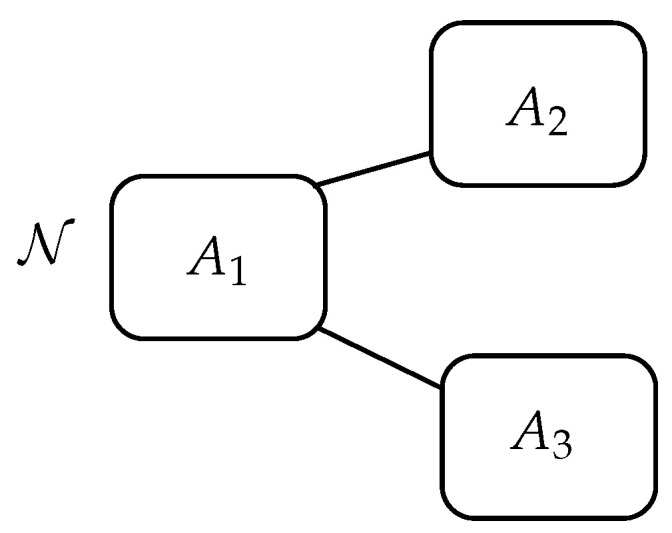
Schematic representation of the interactions and the diffusion of news N among the three agents: Agent 1 interacts with Agents 2 and 3, while Agents 2 and 3 do not interact between themselves.

The first application we present is related to the diffusion of news N in a network with three agents. We suppose that, initially Agent 1 diffuses good news N, and that this agent can interact and be influenced by two other agents, 2 and 3, whop, in turn, do not interact between themselves (see the schematic representation in Figure 1).

We suppose that the various agents communicate in a different way, and that Agents 2 and 3 behave in a different way regarding the diffusion of news from $A_{1}$: agent $A_{2}$ is inclined to accept only good news, whereas $A_{3}$ *prefers* fake news. We also suppose that $A_{3}$ is inclined to change the nature of N and hence he influences Agent 1 to change the nature of N.

We consider the following set of parameters determining the strength of the various mechanisms previously described. The parameters of the inertial terms are $\omega_{1}^{f,g}\;=\;\omega_{2}^{f,g}\;=\;1,\;\omega_{3}^{f,g}\;=\;0.1$, which express the fact that agents $A_{1}$ and $A_{2}$ tends to be more inclined than $A_{3}$ to maintain their perception about N. The parameters for the interactions of the agents are $p_{1,2}^{g}\;=\;1,\;p_{1,3}^{g}\;=\;0.1,\;p_{1,2}^{f}\;=\;0.1,\;p_{1,3}^{f}\;=\;1$ (remember that $p_{i,j}^{f,g}\;=\;p_{j,i}^{f,g},\forall i,j=1,2,3,\;i\neq j$), expressing the fact that $A_{1}$ and $A_{2}$ share basically good news, whereas $A_{1}$ and $A_{3}$ share fake news. The parameters related to the change in the nature of N of the various agents are $\lambda_{1}=\lambda_{2}=0.2$, $\lambda_{3}=1$, meaning that $A_{3}$ easily changes the nature of N when compared to the more conservative agents $A_{1}$ and $A_{2}$. We consider also the following initial conditions: $G_{1}^{0}=1,\;G_{2,3}^{0}=F_{1,2,3}^{0}=0$, so that initially (at t=0) Agent 1 diffuses good news N.

In order to apply rules $\rho_{1}$ and $\rho_{2}$, we take T=60, and the weights in (13) are chosen as $w_{1}^{f}\left(2\right)=0.1,\;w_{1}^{f}\left(3\right)=1,\;w_{1}^{g}\left(2\right)=1,\;w_{1}^{g}\left(3\right)=0.1$ expressing that $A_{1}$ and $A_{2}$ influence themselves above all for good news, whereas $A_{1}$ and $A_{3}$ for fake news. In the case of rule $\rho_{2}$ we also take $w_{\alpha }^{f,g}\left(\alpha \right)=1,\;\alpha =1,2,3$, meaning that each agent attributes a great role to his own perception of N. It is to be expected that the application of the rules can drastically change the way N is finally perceived by the agents, depending on how the various agents are influenced by the others, and hence depending on whether we adopt the rule $\rho_{1}$ or $\rho_{2}$.

**Figure 2:**
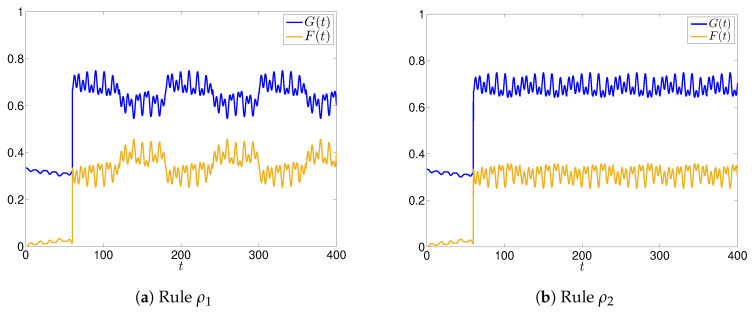
Time evolutions of F(t) and G(t) for the case of 3 agents, and rules $\rho_{1}$ (**a**) and $\rho_{2}$ (**b**).

**Figure 3:**
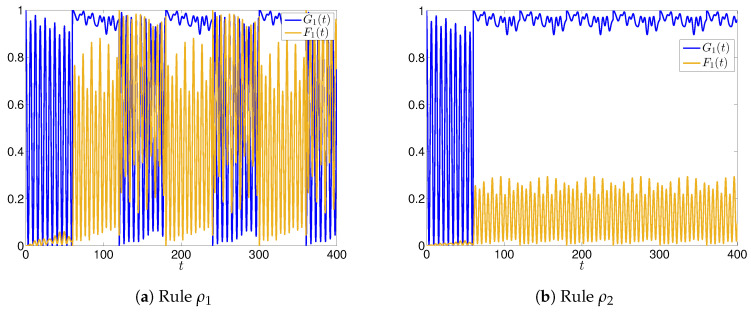
Time evolutions of $F_{1}\left(t\right)$ and $G_{1}\left(t\right)$ for the case of 3 agents, and rules $\rho_{1}$ (**a**) and $\rho_{2}$ (**b**): phases of strong oscillations are more evident in the case of rule $\rho_{1}$.

The time evolution of the main function F(t) and G(t) for the two rules $\rho_{1}$ and $\rho_{2}$ are shown in Figure 2 (we recall from the last remark of Section 2.1 that we have F(t)=1−G(t),∀t≥T.), while in Figure 3 we show the time evolution of the functions $F_{1}\left(t\right)$ and $G_{1}\left(t\right)$, focusing on agent $A_{1}$.

Let us first analyze the case of rule $\rho_{1}$. We notice that in every sub interval [(k−1)T,kT], all the evolutions are periodic with alternating phases in which strong oscillations appear, representing a sort of uncertainty in how news is perceived. The appearance of the oscillations is even more pronounced if we look at the evolution for a specific agent, say $A_{1}$ (the dynamics of other agents, not shown here, exhibit similar oscillations). This is clearly seen by comparing Figure 2 and Figure 3. The main reason for that is due to the way $A_{1}$ interacts with $A_{3}$: in fact, $A_{3}$ receives good news N, and converts the nature of N that becomes fake (high value of $\lambda_{3}$), and induces $A_{1}$ to change the nature of N (high values of $p_{1,3}^{f}$ and $w_{1}^{f}\left(3\right)$). On the other side, $A_{2}$ is inclined to maintain the nature of N, which explains why, despite the phases of oscillations/uncertainty, news N remains on average good (G(t)>F(t),∀t), regardless of the uncertainty induced by $A_{3}$.

On the contrary, by considering the *active* rule $\rho_{2}$, we have that in this case the agents are inclined to be a little bit less influenced by the surrounding agents, since they give a non-zero *weight* to their own perception of news. In fact, we can observe from Figure 2b and Figure 3b that the amplitudes of the oscillations are weakened, and the perception of the goodness of N is higher than that when we adopt rule $\rho_{1}$ case (in the sense that the mean value of G(t) is higher). Moreover, as expected from the application of $\rho_{2}$, the evolution of $G_{1}\left(t\right)$ exhibits a lower uncertainty, this value being very close to the maximum value 1 for t≥T.

**Figure 4:**
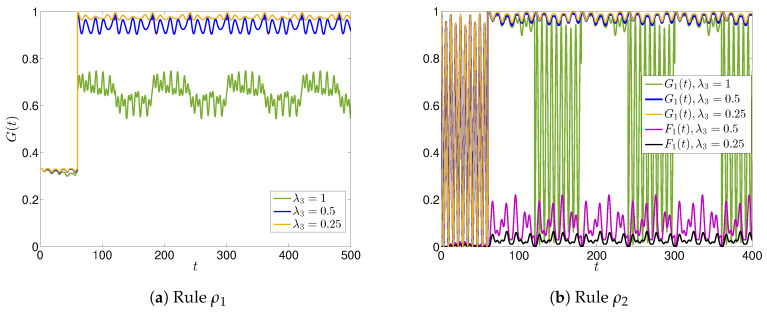
Time evolutions of $G\left(t\right),G_{1}\left(t\right)$ and $F_{1}\left(t\right)$ for the case of three agents for different value of the interaction parameter $\lambda_{3}$, and for rules $\rho_{1}$ (**a**) and $\rho_{2}$ (**b**).

We now present the results obtained by lowering the parameter $\lambda_{3}$ which is responsible for the highly variable nature of N for $A_{3}$ and, as a consequence, for $A_{1}$. In Figure 4, we plot the time evolution of $G\left(t\right),G_{1}\left(t\right)$ and $F_{1}\left(t\right)$ for different values of $\lambda_{3}$ and compare them with the already analyzed case $\lambda_{3}=1$. As expected, moderate low values $\lambda_{3}≲0.5$ determine a more stable time evolution, since $A_{3}$ is less inclined to change the nature of N diffused by $A_{1}$: the news is globally perceived good, as both G(t) and $G_{1}\left(t\right)$ get very close to the maximum value 1.

### 3.2. A Network with Seven Agents

**Figure 5:**
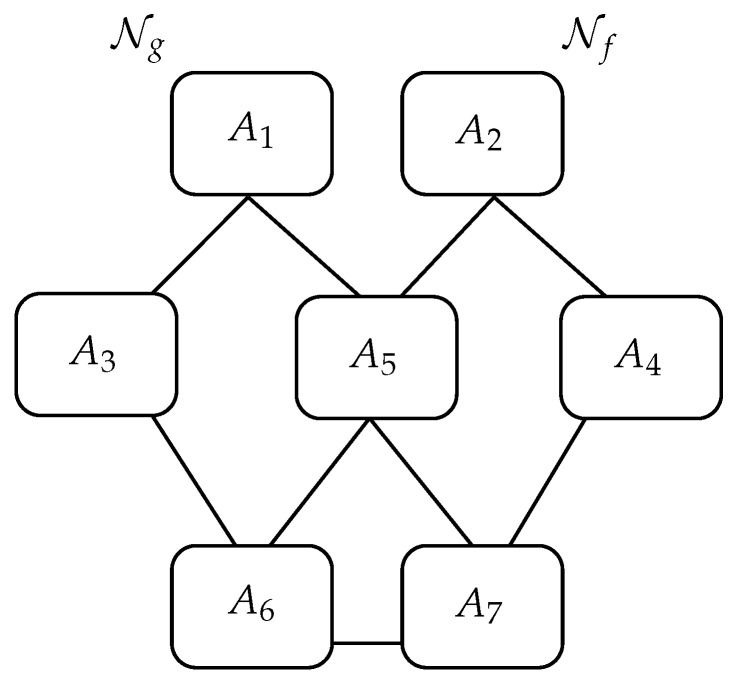
Schematic representation of the network with 7 agents.

The second application we present is the diffusion of news among agents belonging to different levels. These levels could represent (from the highest to the lowest) transmitters verifying the nature of the news with some investigations, inquiries, validations, agents representing some media, and agents which are actually a generic class of final receivers of news (for instance, people getting information from social media, or people using only reliable TV news programs.). A schematic representation of this model and the connections among the agents is shown in Figure 5. The first level is made by two non-interacting main transmitters. We suppose that good news, $N_{g}$ is transmitted by agent $A_{1}$, and fake news, $N_{f}$, by agent $A_{2}$. The second level consists of three agents interacting with the main transmitters, and with the two final receivers: only the agent $A_{5}$ interacts with all the transmitters and receivers. Finally, the third level is made by two receivers communicating between themselves too; therefore, they can influence the perception of news of the other receiver. From the schematic representation of the interaction paths, we can observe that the agent $A_{6}$ (the first receiver) looks more influenced by the agent $A_{1}$ and then by agents $A_{3}$ and $A_{5}$, whereas the $A_{7}$ (the second receiver) by $A_{2}$ and then by agents $A_{4}$ and $A_{5}$: therefore, without considering the mechanisms responsible for the change in news, the two receivers are inclined to perceive $N_{g}$ and $N_{f}$, respectively, as sent by the transmitters, at least if the strength of the various interactions are similar. Of course, the real interesting situation is when some agent changes the reliability of news and how the receivers react accordingly.

The initial conditions for this model are easily written: $G_{1}^{0}=F_{2}^{0}=1$, while $G_{j}^{0}=0$ for j≠1, and $F_{j}^{0}=0$, for j≠2. The parameters $\omega^{f,g}$ of the free dynamics are all taken equal to 0.5, and the other parameters are chosen accordingly to strengthen or weaken the influence of the other mechanisms with respect to the free dynamics.

The interaction parameters are different from zero only for the related agents having connections, as shown in Figure 5, and depend also on the kind of initial news; among all the possible choices, we set $p_{1,3}^{g}=p_{1,5}^{g}=p_{2,4}^{f}=p_{2,5}^{f}=1$, $p_{3,6}^{g}=p_{4,7}^{f}=0.5$, $p_{5,6}^{f}=p_{5,7}^{f}=0.25$, and $p^{f,g}\left(6,7\right)=1$, and of course $p_{i,j}^{f,g}=p_{i,j}^{f,g}$ for all i≠j. The above choices reflect a certain strength between the agents of the first two levels, and between the final receivers which could represent a real situation in which people easily, and very often without care, transmit news or modify it using social media.

In this simulation, we consider the possibility that only the final receivers are able to change the way news is diffused (this could mimic the way the people modify or distort news), and we set $\lambda_{7}=0.05$, whereas $\lambda_{6}$ is varied. Of course, in the case where $\lambda_{6}>\lambda_{7}$, it results that agent $A_{6}$ is more inclined to change the nature of the news $N_{g}$ to fake. The fact that $\lambda_{7}$ is in general much smaller then the others parameters means that agent $A_{7}$ is almost *fair* and essentially communicates news as it is received (no matter good or fake), so that agent $A_{6}$ can be the main party responsible for the alteration of news.

For the application of the rules $\rho_{1},\rho_{2}$ we take as before T=60, and the weights used in (13) are set equal to the related interaction parameters: $w_{i}^{f}\left(j\right)=p_{i,j}^{f},\;w_{i}^{g}\left(j\right)=p_{i,j}^{g}$, and only for rule $\rho_{2}$ we also take $w_{i}^{f,g}\left(i\right)=1,\;\forall \;i$.

**Figure 6:**
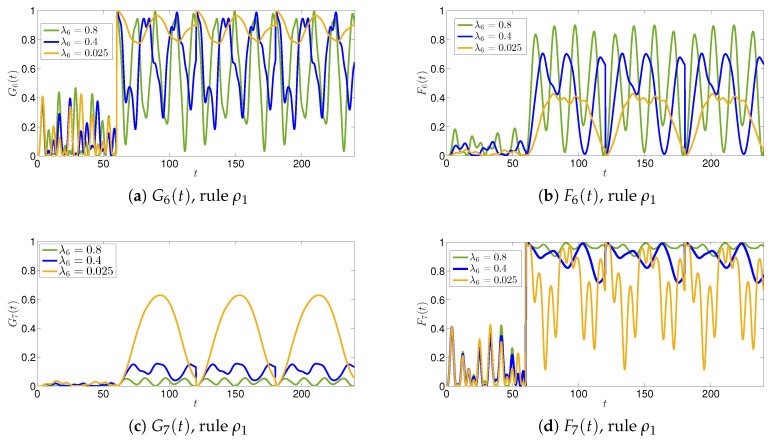
The 7 agents agents model with the application of rule $\rho_{1}$. The evolutions of the functions $G_{6}\left(t\right),F_{6}\left(t\right),G_{7}\left(t\right)$ and $F_{7}\left(t\right)$ are shown for different values of the parameters $\lambda_{6}$. The schematic representation of the interactions between agents are shown in Figure 5.

We first present the results related to rule $\rho_{1}$, shown in Figure 6 the time evolutions of the functions $G_{6}$, $F_{6}$, $G_{7}$ and $F_{7}$ for different values of the parameter $\lambda_{6}$. We can observe that, for $\lambda_{6}=0.025$, there is a different tendency of the agents in changing the way news is transmitted by the main agents $A_{1}$ and $A_{2}$. In fact, after the application of the rule at T=60, we have that $G_{6}$ oscillates very close to the maximum value 1, whereas $F_{6}$ is very low, meaning that agent $A_{6}$ considers news transmitted by $A_{1}$ as good. At the same time, due to the interactions with the agent $A_{6}$ ($p_{6,7}^{f,g}>0$), $A_{7}$ changes its perception of fake news $N_{f}$, and both functions $G_{7}$ and $F_{7}$ show evident oscillations, which express the uncertainty of the agent $A_{7}$.

For $\lambda_{6}=0.4$, we can observe a slightly more stable situation. In fact, despite the fact that the uncertainty of $A_{6}$ increases, we still have $G_{6}\left(t\right)>F_{6}\left(t\right)$, and the uncertainty of $A_{7}$ is significantly decreased as $F_{7}\left(t\right)>G_{7}\left(t\right)$—on average, agents $A_{6}$ and $A_{7}$ consider news as good when it is transmitted by $A_{1}$, and fake when transmitted by $A_{2}$. Increasing $\lambda_{6}$ further has remarkable effects on the uncertainty of agent $A_{6}$, while decreasing that of agent $A_{7}$. In fact, for $\lambda_{6}=0.8$, there is no clear determination of the reliability of the news for agent $A_{6}$, and $G_{6}$ and $F_{6}$ oscillate in all the interval [0,1]. The oscillations of $F_{7}$ and $G_{7}$ are instead damped and $G_{7}\approx 0$, $F_{7}\approx 1$, so that $A_{7}$ perceives the news as fake. The reason for this dynamics is that, for large $\lambda_{6}$, $A_{6}$ is inclined to change the nature of $N_{g}$ to fake, and this reinforces the perception of news by $A_{7}$ as fake.

**Figure 7:**
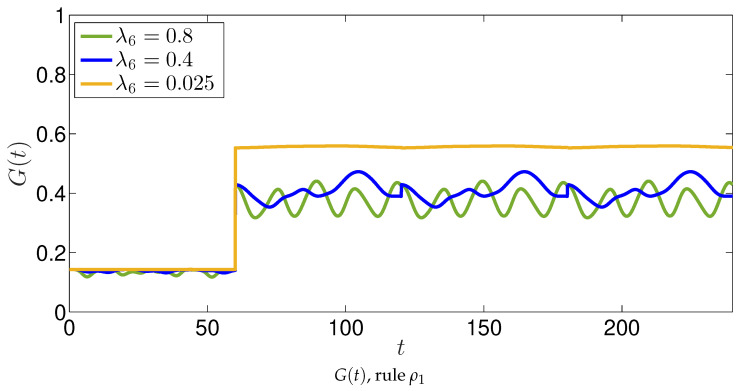
The 7 agents agents model with the application of the rule $\rho_{1}$. The evolution of the function G(t) is shown for different values of the parameters $\lambda_{6}$. The schematic representation of the interactions between agents are shown in Figure 5.

We also show the global function G(t) in Figure 7 for the various $\lambda_{6}$ we have considered (we recall that, after the time *T*, it results in F(t)=1−G(t)). Again, oscillations are wider for larger $\lambda_{6}$, and the overall mean values decrease for increasing $\lambda_{6}$. This is somewhat expected, because the larger $\lambda_{6}$$A_{6}$ is more inclined in changing news from good to fake, so that F(t)>G(t).

**Figure 8:**
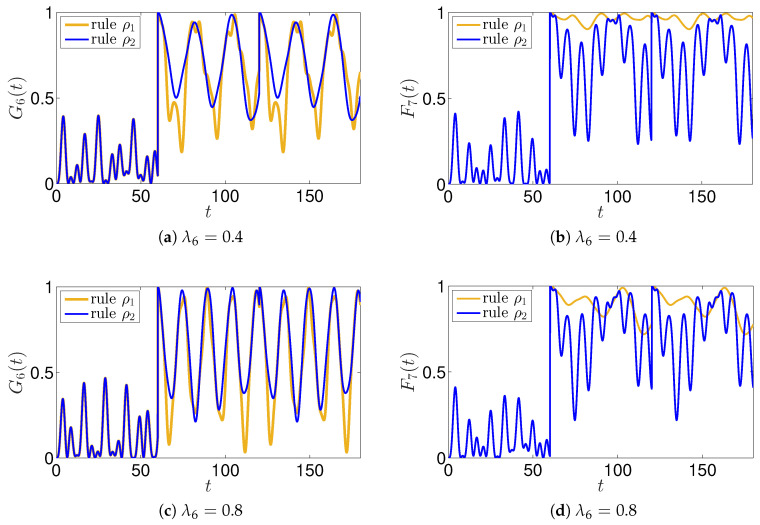
The 7 agents agents model with the application of rules $\rho_{1}$ and $\rho_{2}$. The evolutions of the functions $G_{6}\left(t\right)$ and $F_{7}\left(t\right)$ are shown for different values of the parameters $\lambda_{6}$.

The results concerning the application of rule $\rho_{2}$ are shown in Figure 8, where the functions $G_{6}$ and $F_{7}$ are shown for values $\lambda_{6}=0.8$ and $\lambda_{6}=0.4$, and compared with the results of the rule $\rho_{1}$. The overall outcome is that all the uncertainties are weakened for the agent $A_{6}$, and reinforced for the agent $A_{7}$; nevertheless, we observe that a global uncertainty arises that requires a more deep analysis.

## 4. Conclusions

In this paper, we have shown how a quantum-like approach can be used to describe the spreading of news in a network. In particular, we have considered a system where both good and fake news move. The analysis was based on the use of ladder operators obeying the canonical anti-commutation relations and their related number operators. We have also discussed what happens when some checks (our rule) are applied, from time to time, to the system, and how this modifies the dynamics of the spreading. Incidentally, we do not expect significant changes for larger sizes of networks, at least when compared with those in Figure 5, since each agent could be seen as a cluster of agents with similar behavior, which is very close to the reality, where clusters of people sharing some similar attitude or idea quite easily can be observed.

Our analysis opens the way to many possible applications, from the application of different rules, to the possibility of modeling special classes of agents (e.g., the so-called influencers) in the network. These are only few of the possible extensions of the ideas discussed here.

## Appendix A. (H,ρ)-Dynamics

Let S be our physical system and let $O_{j}$ be a set of *M* commuting self-adjoint operators, needed for the complete description of S, with eigenvectors $\varphi_{\alpha_{n}}^{\left(j\right)}$ and eigenvalues $\alpha_{n}^{\left(j\right)}$:

(A1) $$\left[O_{j},O_{k}\right]=0,\;O_{j}=O_{j}^{†},\;O_{j}\varphi_{n_{j}}^{\left(j\right)}=\alpha_{n_{j}}^{\left(j\right)}\varphi_{n_{j}}^{\left(j\right)},$$

j=1,2,…,M, $n_{j}=1,2,3,\ldots ,N_{j}$, which can be finite or infinite. Let $n=(n_{1},n_{2},\ldots ,n_{M})$ and let

$$\varphi_{n}=\varphi_{n_{1}}^{\left(1\right)}⊗\varphi_{n_{2}}^{\left(2\right)}⊗\cdots ⊗\varphi_{n_{M}}^{\left(M\right)}.$$

Then $\varphi_{n}$ is an eigenstate of all the operators $O_{j}$—i.e.,

(A2) $$O_{j}\;\varphi_{n}=\alpha_{n_{j}}^{\left(j\right)}\;\varphi_{n}.$$

We can safely assume that these vectors satisfy

(A3) $$\langle \varphi_{n},\varphi_{m}\rangle =\delta_{n,m}=\prod_{j=1}^{M}\delta_{n_{j},m_{j}}.$$

The Hilbert space H where S is defined is the closed linear span of all the vectors $\varphi_{n}$. Now, let $H=H^{†}$ be the (time-independent) self-adjoint Hamiltonian of S. This means that, in the absence of any other information, the wave function Ψ(t) describing S at time *t* evolves according to the Schrödinger equation $i\dot{\Psi }\left(t\right)=H\Psi \left(t\right)$, where $\Psi \left(0\right)=\Psi_{0}$ describes the initial status of S. The formal solution of the Schrödinger equation in t∈[0,τ[, for a fixed τ>0, is $\Psi_{0}\left(t\right)=\exp\left(-iHt\right)\Psi_{0}$. Now let ρ be our *rule*—i.e., a set of conditions mapping, at a certain time, any input vector $\Psi_{0}\left(\tau \right)\in H$ in a new vector $\Psi_{1}\in H$—and with a synthetic notation we will simply write $\Psi_{1}=\rho \left(\Psi_{0}\left(\tau \right)\right)$. Then, in the new time interval t∈[0,τ], the new vector $\Psi_{1}$ evolves according to the Schrödinger evolution, $\Psi_{1}\left(t\right)=\exp\left(-iHt\right)\Psi_{1}$, and at time τ we map $\Psi_{1}\left(\tau \right)$ into a new state $\Psi_{2}$. The procedure can continue for more iterations k=1,2,⋯. Now let *X* be a generic bounded operator on H. For instance, in Section 2 we have considered *X* as the number operators related to good and fake news.

**Definition** **A1.**
*The sequence of functions*

(A4) $$x_{k}\left(t\right):=\langle \Psi_{k},X\left(t\right)\Psi_{k}\rangle ,$$

*for t∈[0,τ] and $k\in N_{0}$, is called the (H,ρ)-induced dynamics of the operator X.*

We refer to [14] (and to the references therein) for more details of the (H,ρ)-induced dynamics. Here we only observe that, using $X\left(t\right)=(x_{1}\left(t\right),x_{2}\left(t\right),x_{3}\left(t\right),\ldots )$, it is possible to define a function of time in the following way:

(A5) $$\tilde{X}\left(t\right)=\left\{\begin{array}{cc} x_{1}\left(t\right),\;t\in [0,\tau [ & \\ x_{2}\left(t-\tau \right),\;t\in [\tau ,2\tau [ & \\ x_{3}\left(t-2\tau \right),\;t\in [2\tau ,3\tau [ & \\ \ldots \end{array}\right.$$

It is clear that $\tilde{X}\left(t\right)$ may have discontinuities in kτ, for k∈N. In [14] ,we have discussed conditions for $\tilde{X}\left(t\right)$ to admit some asymptotic value or to be periodic, and some related notions of *equilibria* for (H,ρ)-dynamics.
